# Taurine bolsters adoptive T cell therapy efficacy against hepatocellular carcinoma by reprogramming T cell function

**DOI:** 10.1007/s00262-026-04503-y

**Published:** 2026-08-06

**Authors:** Zhiming Zhang, Siwei Li, Mengjie Li, Li Gong, Xinyu Lu, Weikang Xu, An-an Chen, Yiyue Wang, Changsheng He, Guoheng Mo, Yuting Xie, Si-an Yue, Lei Huang, Chou Yang, Jinlin Hou, Wei Zhu

**Affiliations:** 1 Department of Infectious Diseases, Nanfang Hospital, Southern Medical University; State Key Laboratory of Multi-organ Injury Prevention and Treatment; Key Laboratory of Infectious Diseases Research in South China (Southern Medical University), Ministry of Education; Guangdong Provincial Key Laboratory for Prevention and Control of Major Liver Diseases; Guangdong Provincial Clinical Research Center for Viral Hepatitis; Guangdong Institute of Hepatology, Guangdong Provincial Research Center for Liver Fibrosis Engineering and Technology, Guangzhou, 510515 China; 2 Department of Gastroenterology, Integrative Microecology Clinical Center, Shenzhen Key Laboratory of Gastrointestinal Microbiota and Disease, Shenzhen Clinical Research Center for Digestive Disease, Shenzhen Hospital, Southern Medical University, Shenzhen, 518101 China; 3 Department of General Practice, Shenzhen Hospital of Southern Medical University, Shenzhen, 518100 China; 4 Experimental Animal Center, Nanfang Hospital, Southern Medical University, Guangzhou, 510515 China; 5 Department of Gastroenterology, The Second Affiliated Hospital of Guangzhou Medical University, Guangzhou, 510220 China; 6 Translational and Clinical Research Institute, FMS, Newcastle University, Newcastle Upon Tyne, NE4 4HH UK

**Keywords:** Taurine, Adoptive T cell, Hepatocellular carcinoma, SLC6A6

## Abstract

Adoptive T cell therapy for advanced hepatocellular carcinoma (HCC) is limited by the immunosuppressive tumor microenvironment, which impairs T-cell function. Overcoming this immunosuppression and restoring T-cell functionality are crucial for improving therapeutic efficacy. Here, we investigated the role of taurine in regulating alpha-fetoprotein (AFP)-specific T-cell receptor-engineered T cells (TCR-T) function. Metabolomic profiling revealed that intracellular taurine levels declined during the early phase of T-cell activation, while expression of the taurine transporter SLC6A6 increased over time. In addition, taurine abundance was lower in HCC tissues than in normal liver tissues in vivo, suggesting that taurine availability is reduced during both T-cell activation and tumor progression. Functionally, taurine supplementation enhanced the cytotoxic activity, persistence, and intratumoral infiltration of AFP-specific TCR-T, while reducing exhaustion and apoptosis after repetitive tumor stimulation. In contrast, taurine-limited conditions impaired T-cell proliferation and survival, attenuated antitumor activity in vitro and in vivo, and promoted an exhaustion-prone phenotype. Taurine supplementation also partially restored the function of β-alanine-preconditioned T cells. Mechanistically, transcriptomic and proteomic analyses identified PI3K-AKT signaling as a key pathway associated with taurine-mediated T-cell regulation. Taurine-limited T cells showed reduced AKT phosphorylation and developed a metabolically stressed state characterized by increased mitochondrial respiration, glycolytic activity, reactive oxygen species production and mitochondrial structural abnormalities during activation. Together, these findings identify taurine availability as an important metabolic regulator of engineered T-cell fitness and support taurine-based metabolic conditioning as a potential strategy to improve TCR-T therapy for HCC.

**Supplementary Information:**

The online version contains supplementary material available at https://doi.org/10.1007/s00262-026-04503-y.

## Introduction

Hepatocellular carcinoma (HCC) constitutes the majority of primary liver cancers and ranks as the third leading cause of cancer-related mortality [1]. Current therapeutic strategies for HCC primarily include local ablation, surgical resection, and liver transplantation [2]. However, therapeutic options for advanced HCC remain limited. Although current first-line systemic therapies have improved clinical outcomes, recent phase III trials have reported a median overall survival of merely 16.4–19.2 months [3, 4 ], highlighting the persistent unmet clinical demand for innovative and more effective treatments in HCC patients.

Adoptive T cell therapy (ACT), particularly with engineered T cells such as T-cell receptor-engineered T cells (TCR-T), has emerged as a highly promising strategy in cancer treatment. While ACT demonstrates robust antitumor activity in hematological malignancies [5, 6 ], its efficacy in solid tumors, including HCC, remains unsatisfactory [7, 8 ]. This limitation is partly attributed to the immunosuppressive tumor microenvironment (TME) of solid tumors, which is characterized by abnormal vasculature, hypoxia, and diverse immunosuppressive cell populations [9, 10 ]. In addition, heightened tumor metabolism and restricted nutrient availability within the TME can impair the survival, persistence, and effector function of tumor-infiltrating T cells [11, 12 ]. Accordingly, improving the metabolic fitness of therapeutic T cells has become an important strategy for enhancing ACT efficacy in solid tumors.

Taurine, a sulfur-containing amino acid that is not incorporated into proteins, is abundantly present in tissues including the brain, heart, and skeletal muscles [13]. It can be obtained from dietary sources or synthesized endogenously from cysteine through the sequential actions of cysteine dioxygenase and cysteine sulfinate decarboxylase [14, 15 ]. Intracellular taurine levels are regulated primarily by transporter-mediated uptake, with the SLC6A6-encoded taurine transporter playing a central role. Taurine has been implicated in a wide range of physiological processes, including osmotic homeostasis [16], protection against oxidative stress [17, 18 ], maintenance of mitochondria integrity [19, 20 ], and mitigation of aging processes [21]. Notably, taurine deficiency in T cells has been associated with increased apoptosis and impaired proliferation upon activation [22, 23 ], underscoring its critical role in T cell-mediated immunity. Recent studies have also highlighted that tumor cells compete with immune cells for taurine by overexpressing *SLC6A6*, depleting this amino acid in the TME and driving exhaustion of tumor-infiltrating CD8^+^ T cells [24]. Taurine supplementation has been shown to reverse T-cell exhaustion, enhance CD8^+^ T cell antitumor responses, and synergize with PD-1 blockade therapy [24, 25 ]. Nevertheless, the role of taurine availability in ACT, particularly in engineered T cells targeting solid tumors such as HCC, remains insufficiently defined.

AFP was selected as the target antigen because of its well-established role in HCC surveillance and diagnosis [26]. Moreover, the HLA-A*02:01-restricted AFP-derived peptide represents a targetable antigenic complex for TCR-T therapy and AFP-specific TCR-T cells (AFP-TCR-T) have been functionally validated in our previous studies [27]. Therefore, in this study, we used AFP-TCR-T as a clinically relevant antigen-specific model to investigate how taurine availability regulates engineered T-cell function in HCC. We first characterized taurine dynamics during T-cell activation and in tumor tissues and then examined the effects of taurine supplementation and taurine-limited conditions on T-cell proliferation, survival, exhaustion, and antitumor activity in vitro and in vivo. In addition, we explored the signaling and metabolic changes associated with taurine-mediated regulation of T-cell function. Our results show that taurine availability is closely linked to the functional fitness of engineered T cells, and taurine supplementation enhances the persistence and antitumor efficacy of AFP-TCR-T [27, 28 ] in vitro and in vivo, whereas taurine-limited conditions impaired these functions. These findings support taurine-based metabolic conditioning as a potential strategy to improve ACT for HCC.

## Materials and methods

### Mice

Female B-NDG and C57BL/6 J mice (6–8 weeks old) were procured from BesTest Biotech Company (Zhuhai, China). Mice were housed under specific pathogen-free conditions with ad libitum access to water and standard irradiated rodent chow supplied by the Guangdong Medical Laboratory Animal Center. All animal experiments were approved by the Institutional Animal Care and Use Committee of Nanfang Hospital (IACUC-LAC-20230808-001).

### Cell line

HepG2 (HLA-A2^+^/AFP^+^) cells were sourced from iCell Bioscience Inc. (Shanghai, China) and maintained in DMEM (Gibco) supplemented with 10% unheated fetal bovine serum (Gibco), 100 U/mL penicillin (Gibco), and 0.1 mg/mL streptomycin (Gibco) at 37 ℃ with 5% CO_2_.

### Generation of recombinant lentivirus

The sequence of AFP-specific TCR was the same as previously mentioned [29]. Lentivirus expressing AFP-specific TCR was prepared and titrated by Genscript Biotech Company (Nanjing, China).

### Transduction and ex vivo expansion of human T cells

Human T cells were isolated by negative selection from PBMCs obtained from three healthy male volunteers aged 25–35 years and activated with a CD3/CD28 tetrameric antibody complex (Stemcell Technologies, Canada) for 3 days before lentiviral transduction. The antibody complex was removed 5 days after transduction. Cells were cultured in RPMI 1640 medium supplemented with 10% heat-inactivated fetal bovine serum, 10 ng/mL recombinant human IL-2 (PeproTech, USA), 100 U/mL penicillin, 0.1 mg/mL streptomycin, 10 mM HEPES, 1× MEM non-essential amino acids, and 55 μM 2-mercaptoethanol (all from Gibco, unless specified). Cell density was maintained at 0.5–2.5 × 10⁶ cells/mL.

### Plasmid-induced autochthonous HCC model

A plasmid-induced autochthonous HCC model was established in C57BL/6 J mice using the Sleeping Beauty transposon system and hydrodynamic tail vein injection as previously described [30]. Briefly, pT3-EF1aH N90-β-catenin (Addgene plasmid #86499), pT3-EF1aH c-Met (Addgene plasmid #86498), and pCMV/SB10 (Addgene plasmid #24551) were obtained from Addgene (Watertown, MA, USA). The plasmids were diluted in sterile PBS and injected into the lateral tail vein within 5-8 s at a volume equivalent to approximately 10% of body weight. Mice were euthanized at the indicated time points, and liver tumor tissues together with normal liver tissues from control mice were collected for subsequent analyses.

### Flow cytometry analysis and antibodies

Single-cell suspensions were prepared for flow cytometry analysis. For surface staining, cells were incubated with specific antibodies for 15 min at room temperature. For apoptosis detection, cells were stained in Annexin V Binding Buffer (BioLegend, USA) for 30 min at 4 °C. For intracellular transcription factor staining, cells were fixed and permeabilized using the Foxp3/Transcription Factor Staining Buffer Set (eBioscience, USA) according to the manufacturer’s instructions, followed by staining with indicated antibodies. After washing, samples were acquired on a CytoFLEX flow cytometer (Beckman Coulter) and analyzed using FCS Express VI software. Antibodies are listed in the Supplementary Methods.

### In vitro repetitive coculture assay

HepG2 cells (1 × 10^5^ per well) were seeded in 96-well plates for 24 h before addition of AFP-TCR-T cells at an effector-to-target ratio of 0.5:1 in the presence of recombinant human IL-2 (10 ng/mL). For β-alanine treatment, AFP-TCR-T cells were pretreated with 100 mM β-alanine, a taurine analog used as a pharmacological tool to establish taurine-limited conditions, for 72 h before coculture, and 100 mM β-alanine was maintained during subsequent coculture rounds. For taurine supplementation experiments, taurine was added at the indicated concentrations of 10, 20, 40, and 80 mM during the dose-response assay, and 40 mM taurine was used for subsequent pretreatment and rescue experiments. In taurine-treated groups, taurine was maintained in the coculture medium throughout the indicated coculture rounds. T cells were harvested every 36h and transferred to freshly seeded HepG2 cells for three consecutive rounds. Three rounds of repetitive coculture were performed to model repeated tumor antigen exposure and to evaluate the sustained cytotoxic function and durability of AFP-TCR-T cells under chronic tumor challenge conditions. For pharmacological modulation of AKT signaling, β-alanine-pretreated AFP-TCR-T cells were cocultured with HepG2 cells in the presence of SC79 (1 μM), a pharmacological AKT activator, whereas taurine-pretreated AFP-TCR-T cells were cocultured with MK2206 (4 μM), a selective allosteric AKT inhibitor.

### Lactate dehydrogenase (LDH) assay and enzyme-linked immunosorbent assay (ELISA)

Culture supernatants were collected after each round of coculture. Cytotoxicity was quantified using an LDH release assay (Promega, USA) according to the manufacturer’s instructions. To calculate target-cell cytotoxicity, HepG2 target cells alone, AFP-TCR-T effector cells alone, culture medium alone, and lysed HepG2 target cells were included to determine target-cell spontaneous release, effector-cell spontaneous release, medium background, and target-cell maximum LDH release, respectively. After background correction, cytotoxicity was calculated as: [(experimental LDH release − effector-cell spontaneous release − target-cell spontaneous release)/(target-cell maximum release − target-cell spontaneous release)] × 100. Human IFN-γ and TNF-α levels were measured by ELISA (BioLegend), according to the manufacturers’ instructions.

### Construction of xenograft models, adoptive T cell transfer, and analysis of tumor-infiltrating T cells

Subcutaneous HepG2 xenografts were generated in B-NDG mice by right-flank injection of 7.5 × 10^6^ HepG2 cells. Mice received intravenous AFP-TCR-T cells (3-5 × 10^6^ per mouse) plus human IL-2 (50,000 U). Tumor volume was monitored three times weekly and calculated as 1/2 (length × width × height). Tumor-infiltrating T cells were analyzed by flow cytometry after enzymatic dissociation.

### T-cell proliferation assay

T-cell proliferation was assessed by CFSE dilution using the CellTrace™ CFSE Cell Proliferation Kit (Thermo Fisher Scientific, USA). Labeled cells were activated with the CD3/CD28 tetrameric antibody complex for 7 days before flow cytometric analysis.

### Western blot analysis

Cell lysates were prepared in loading buffer, denatured, separated by SDS-PAGE, and transferred to PVDF membranes (Millipore, USA). Membranes were incubated overnight at 4 °C with primary antibodies against SLC6A6 (1:1000, ab196821, Abcam), phospho-AKT (Thr308) (1:1000, 4056 T, Cell Signaling Technology), total AKT (1:1000, #4691, Cell Signaling Technology), or anti-β-actin (1:1000, 4970L, Cell Signaling Technology), followed by HRP-conjugated secondary antibodies for 1h at room temperature. Protein bands were visualized using an ECL reagent (Beyotime, China) and imaged with a UVP BioSpectrum AC system.

### Mitochondrial mass, membrane potential, and intracellular ROS detection

Cells were stained with MitoTracker Deep Red (10 nM) or MitoTracker Green (25 nM, Thermo Fisher Scientific) for 20 min at 37 °C in PBS to assess mitochondrial membrane potential and mitochondrial mass, respectively. Intracellular ROS levels were assessed using DCFH-DA (Beyotime Biotechnology), a cell-permeable fluorescent probe that is deacetylated intracellularly and oxidized to fluorescent DCF in the presence of ROS. Therefore, DCFH-DA fluorescence was used as an indicator of overall intracellular oxidative stress. Cells were incubated with DCFH-DA for 20 min at 37 °C in the dark. After washing, samples were analyzed by flow cytometry and processed using FCS Express VI software. All experiments were independently repeated at least twice.

### Seahorse metabolic flux analysis

Oxygen consumption rate (OCR) and extracellular acidification rate (ECAR) were measured using the Seahorse XF-96 Extracellular Flux Analyzer (Agilent Technologies, USA). For OCR analysis, cells were incubated in XF DMEM supplemented with 10 mM glucose and 2 mM glutamine, with sequential injection of oligomycin (2 μM), FCCP (2 μM), rotenone (0.5 μM), and antimycin A (0.5 μM). For ECAR analysis, cells were incubated in glucose-free Seahorse XF medium containing 2 mM glutamine, followed by sequential injection of glucose (10 mM), oligomycin (1 μM), and 2-deoxy-D-glucose (50 mM). Metabolic parameters were calculated using Wave software (Agilent Technologies).

### Transmission electron microscope analysis

Cells were fixed in 2.5% glutaraldehyde in 0.1 M sodium cacodylate buffer (pH 7.4) at 4 °C overnight. Samples were post-fixed with 1% osmium tetroxide for 1h at room temperature, dehydrated through a graded ethanol series, and embedded in epoxy resin. Ultrathin Sects. (70 nm) were cut and stained with 2% uranyl acetate followed by lead citrate for contrast enhancement. Images and digital images were acquired and captured using a transmission electron microscope (Hitachi HT-7800) operated at 80 kV. Mitochondrial area was calculated by ImageJ (v1.53e).

### Untargeted metabolomics analysis

The metabolomics of T cells was analyzed by Biotree Biomedical Technology Company (Shanghai, China). Detailed procedures for metabolomics data processing and analysis are provided in Supplementary methods.

### Targeted metabolomics for taurine

For targeted taurine extraction, cell pellets (1 × 10⁷ cells) or frozen tissues (20-50 mg) were extracted in pre-chilled methanol. For tissue samples, frozen tumor or normal liver tissues were immersed in pre-chilled methanol and disrupted by repeated freeze–thaw cycles and sonication on ice. The extracts were then subjected to protein precipitation at − 40 °C and centrifugation at 4 °C. Supernatants were filtered through 0.22 μm PVDF filters and subjected to LC–MS analysis. Taurine quantification was performed using a Prelude SPLC system coupled to a TSQ Quantiva™ mass spectrometer (Thermo Fisher Scientific). Samples were separated on a Waters Acquity UPLC BEH Amide (2.1 × 100 mm, 1.7 μm) at 40 ℃ using mobile phases A (0.1% formic acid/water) and mobile phase B (0.1% acetonitrile). Blank runs were included to monitor carryover.

### RNA sequencing and analysis

Total RNA was isolated and purified using TRIzol reagent (Invitrogen, USA) following the manufacturer’s procedure. Following RNA sequencing and analysis was performed by Singleron Biotechnology Company (Nanjing, China). Detailed procedures for data processing and analysis are provided in Supplementary methods.

### Proteomic analysis

The proteomic analysis of T cells was analyzed by Metware Metabolism Company (Wuhan, China). Detailed procedures for data processing and analysis are provided in Supplementary methods.

### Statistical analysis

Statistical analysis were performed using GraphPad Prism software (version 9.5.1). Differences between two groups were assessed using two-tailed Student’s t tests. For comparisons among three or more groups, two-way analysis of variance (ANOVA) was applied, while repeated-measures ANOVA was used to analyze tumor volume growth curves over time. A *P* value of less than 0.05 was considered statistically significant. For in vivo experiments, n indicates the number of individual mice per group. For in vitro assays, data are shown from one representative experiment with technical replicates, and each assay was independently repeated with consistent trends. For Western blot analyses, representative blots from independent experiments with consistent results are shown; grayscale intensity ratios were calculated from the displayed blot for descriptive purposes, and no statistical comparison was performed.

### Research checklist

We used the ARRIVE reporting guideline [31] to draft this manuscript, and the ARRIVE reporting checklist was included in supplementary materials.

## Results

### Intracellular taurine decreases upon T-cell activation and is reduced in tumor tissues

**Fig. 1:**
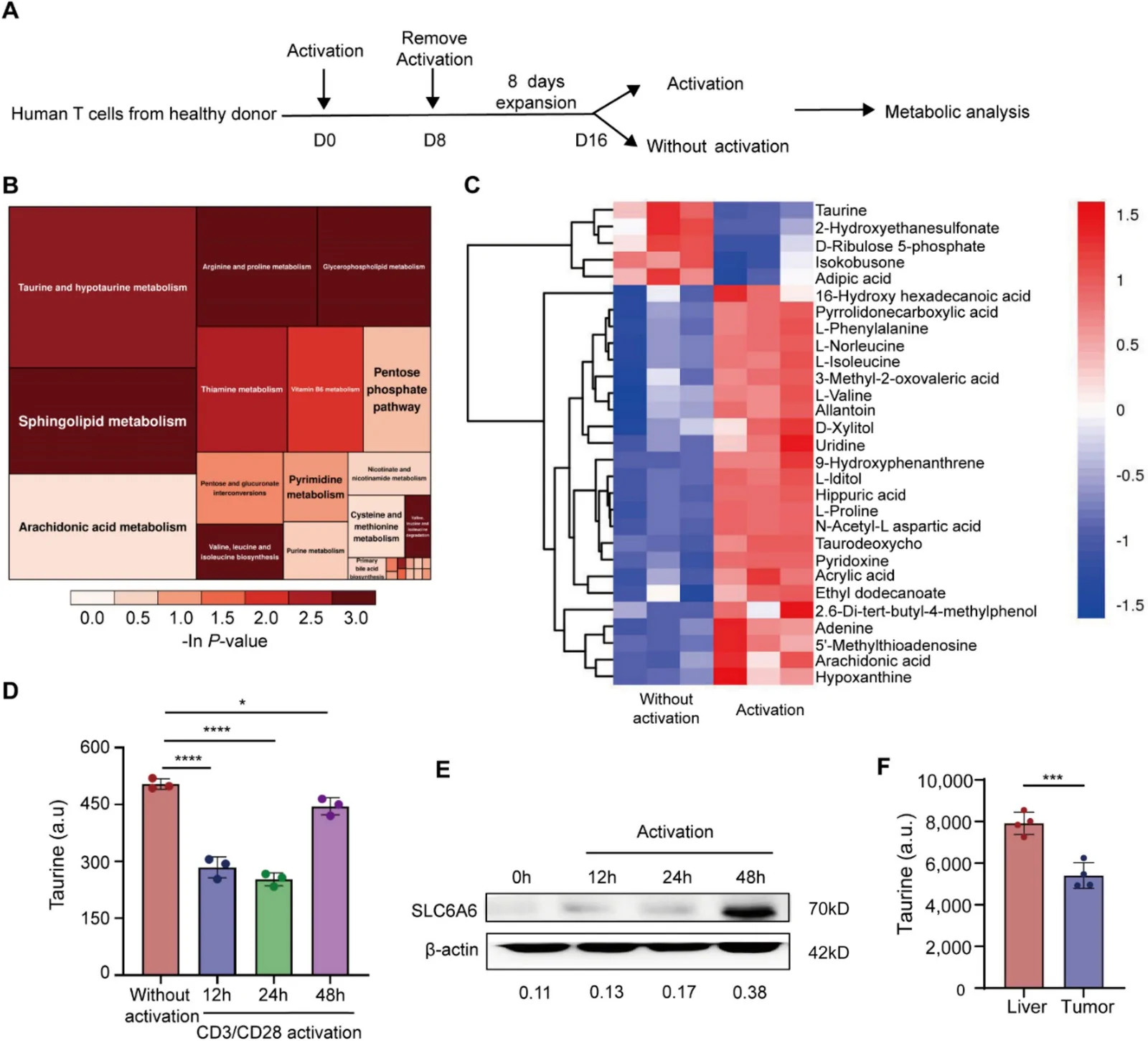
Intracellular taurine decreases upon T-cell activation and is reduced in tumor tissues. **A** The schematic displaying the metabolic analysis of human T cells with or without CD3/CD28 activation. Briefly, T cells were isolated from healthy donor and expanded for 16 days ex vivo. Then, the T cells were activated by CD3/CD28 or not for 48 h and followed by metabolite analysis. **B** The treemap displaying the pathway enrichment analysis of differential metabolites between 48 h-activated and non-activated T cells. **C** The heatmap illustrating metabolite alterations detected by negative-ion mode analysis in T cells with or without 48 h CD3/CD28 activation. **D** The bar graph showing time-dependent reduction of intracellular taurine levels measured by LC–MS during 12 h, 24 h, and 48 h CD3/CD28 activation (*n* = 3). **E** Western blot analysis of SLC6A6 and β-actin expression in T cells without or after 12 h, 24 h, 48 h CD3/CD28 activation. Relative SLC6A6 expression quantified as the gray value ratio of SLC6A6 to β-actin is shown. **F** The bar graph showing taurine levels in tumor tissues from a murine autochthonous HCC model and normal liver tissues from control mice, as measured by LC–MS (*n* = 4). Data were shown as mean ± SEM, *P < 0.05, ****P < 0.0001

To investigate metabolite alterations during T-cell activation, we analyzed the metabolomic profiles of human T cells with or without CD3/CD28 stimulation. T cells isolated from healthy human donors were expanded ex vivo for 16 days and then subjected to CD3/CD28 activation before metabolite analysis (Fig. 1A). Principal component analysis (PCA) revealed a distinct segregation of activated T cells versus non-activated along PC1 (Fig. S1A). Pathway analysis revealed significant enrichment of taurine and hypotaurine metabolism in the negative-ion LC–MS dataset (Fig. 1B and Fig. S1C-D). Metabolite profiling further showed reduced levels of taurine and its precursor 2-hydroxyethanesulfonate, accompanied by accumulation of taurodeoxycholate, after 48 h of CD3/CD28 stimulation (Fig. 1C and Fig. S1B). To validate the untargeted metabolomics data, intracellular taurine levels were measured by LC–MS. Taurine abundance decreased at 12–24 h after activation, confirming an early decline during T-cell activation, but partially recovered between 24 and 48 h (Fig. 1D). This recovery coincided with the progressive upregulation of SLC6A6, which reached its highest level at 48 h (Fig. 1E), suggesting a compensatory increase in taurine uptake capacity during the later phase of T-cell activation. Moreover, taurine levels were significantly higher in normal liver tissues than in HCC tissues in a plasmid-induced autochthonous HCC model (Fig. 1F). This finding is consistent with recent evidence that cancer cells can compete with CD8^+^ T cells for taurine through SLC6A6-mediated uptake, thereby impairing antitumor T-cell immunity [24]. Collectively, these findings indicate that intracellular taurine homeostasis is dynamically regulated during T-cell activation and may be relevant to T-cell function in the tumor microenvironment.

### Taurine limitation compromises T-cell proliferation and weakens TCR-T antitumor function

**Fig. 2:**
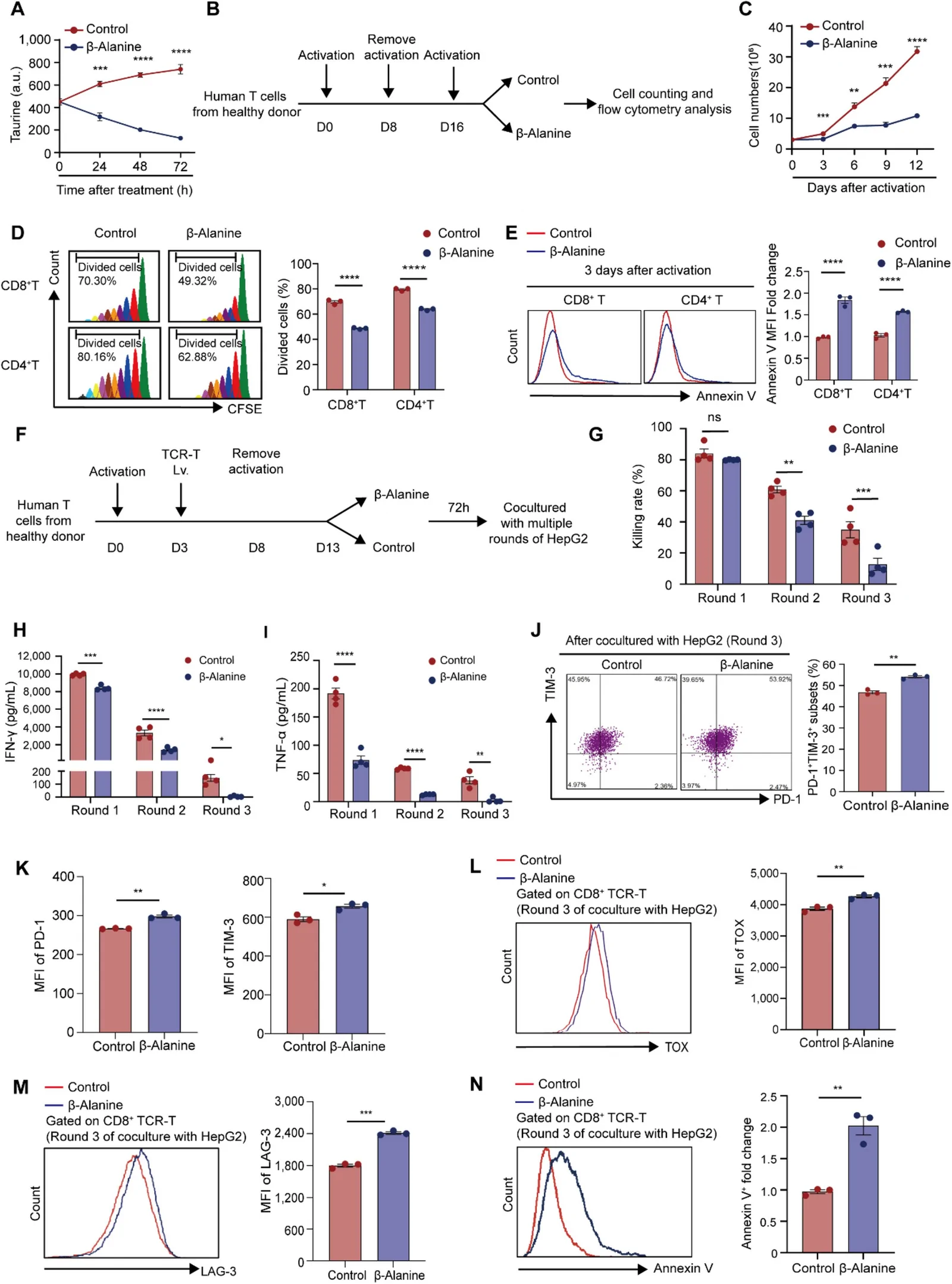
Taurine limitation compromises T-cell proliferation and weakens TCR-T antitumor function. **A** The line graph showing intracellular taurine levels in β-alanine-treated versus untreated T cells by LC–MS (*n* = 3). **B** The process of human T-cell expansion and β-alanine treatment. Briefly, human T cells were isolated from healthy donors and expanded for 16 days ex vivo. Then, the T cells were activated by CD3/CD28 with or without β-alanine treatment. Cells proliferation and apoptosis were monitored by cell counting and flow cytometry detection. **C** The line graph showing the T-cell proliferation with or without β-alanine treatment during CD3/CD28 activation (*n* = 3). **D** Flow cytometry histograms showing CFSE dilution profiles in CD4^+^ and CD8^+^ subsets after 7-day activation. Bar graphs quantify divided cell proportions (*n* = 3). **E** Flow cytometry histograms and the bar graph showing Annexin V MFI fold changes in CD4^+^ and CD8^+^ T cells after 72 h CD3/CD28 activation (*n* = 3). **F** The schematic of AFP-TCR-T generation and β-alanine pretreatment protocol for tumor challenge experiments. **G** The bar graph tracking cytotoxic activity of AFP-TCR-T with or without β-alanine pretreatment through multiple tumor challenge cycles (*n* = 4). **H**-**I** Bar graph showing the supernatant IFN-γ (**H**) and TNF-α (**I**) levels after 36h coculture of HepG2 and AFP-TCR-T with or without β-alanine pretreatment (*n* = 4). **J** Representative flow cytometry plots and quantification of PD-1^+^TIM-3+ subsets in CD8^+^ AFP-TCR-T with or without β-alanine pretreatment after three rounds of coculture with HepG2 cells (*n* = 3) **K** The bar graphs showing mean fluorescence intensity (MFI) of PD-1 and TIM-3 in CD8^+^ AFP-TCR-T with or without β-alanine pretreatment after three rounds of coculture with HepG2 cells (*n* = 3). **L**-**N** Representative histogram overlays and quantification of MFI of TOX (**L**), LAG-3 (**M**), and Annexin V (**N**) in gated CD8^+^ AFP-TCR-T with or without β-alanine pretreatment after round 3 coculture with HepG2 cells (*n* = 3). Data were shown as mean ± SEM, *P < 0.05, **P < 0.01, ***P < 0.001, ****P < 0.0001, NS not significant

To assess the potential role of taurine in T cell biology, we used β-alanine as a pharmacological approach to model taurine-limited conditions during T-cell activation [32, 33 ]. Intracellular taurine levels were markedly reduced in T cells following 24, 48, and 72 h of β-alanine treatment (Fig. 2A). To assess the impact of taurine deficiency in activated T cells, ex vivo-expanded T cells from healthy donors were stimulated with CD3/CD28 in the presence or absence of β-alanine (Fig. 2B). β-Alanine markedly suppressed T-cell expansion, resulting in an approximately threefold reduction in total cell numbers by day 12 after activation (Fig. 2C). Flow cytometric analysis of CFSE-labeled T cells further showed a pronounced reduction in cell division in both CD8^+^ and CD4^+^ T-cell subsets in the β-alanine-treated group (Fig. 2D). In parallel, Annexin V staining revealed elevated apoptosis in both CD8^+^ and CD4^+^ T cells under taurine-deficient conditions (Fig. 2E). Together, these results demonstrate that taurine deficiency compromises both the proliferation capacity and survival of activated T cells.

To model taurine deficiency in engineered T cells, β-alanine-pretreated AFP-TCR-T cells were subjected to serial coculture with HepG2 cells (Fig. 2F). LDH release assays showed comparable cytotoxicity during the first round of coculture, but significantly reduced tumor cell killing during the second and third rounds (Fig. 2G). Consistently, secretion of IFN-γ and TNF-α was markedly decreased throughout serial coculture (Fig. 2H-I). After three rounds of coculture, β-alanine-pretreated AFP-TCR-T showed modest but consistent increases in exhaustion-associated markers, including PD-1, TIM-3, LAG-3, and TOX together with increased Annexin V staining in gated CD8^+^ TCR-T cells (Fig. 2J-N). These phenotypic changes were interpreted together with the impaired serial cytotoxicity and reduced cytokine secretion observed under taurine-limited conditions.

### Taurine supplementation enhances TCR-T anti-HCC function in a concentration-dependent manner and reduces exhaustion and apoptosis

**Fig. 3:**
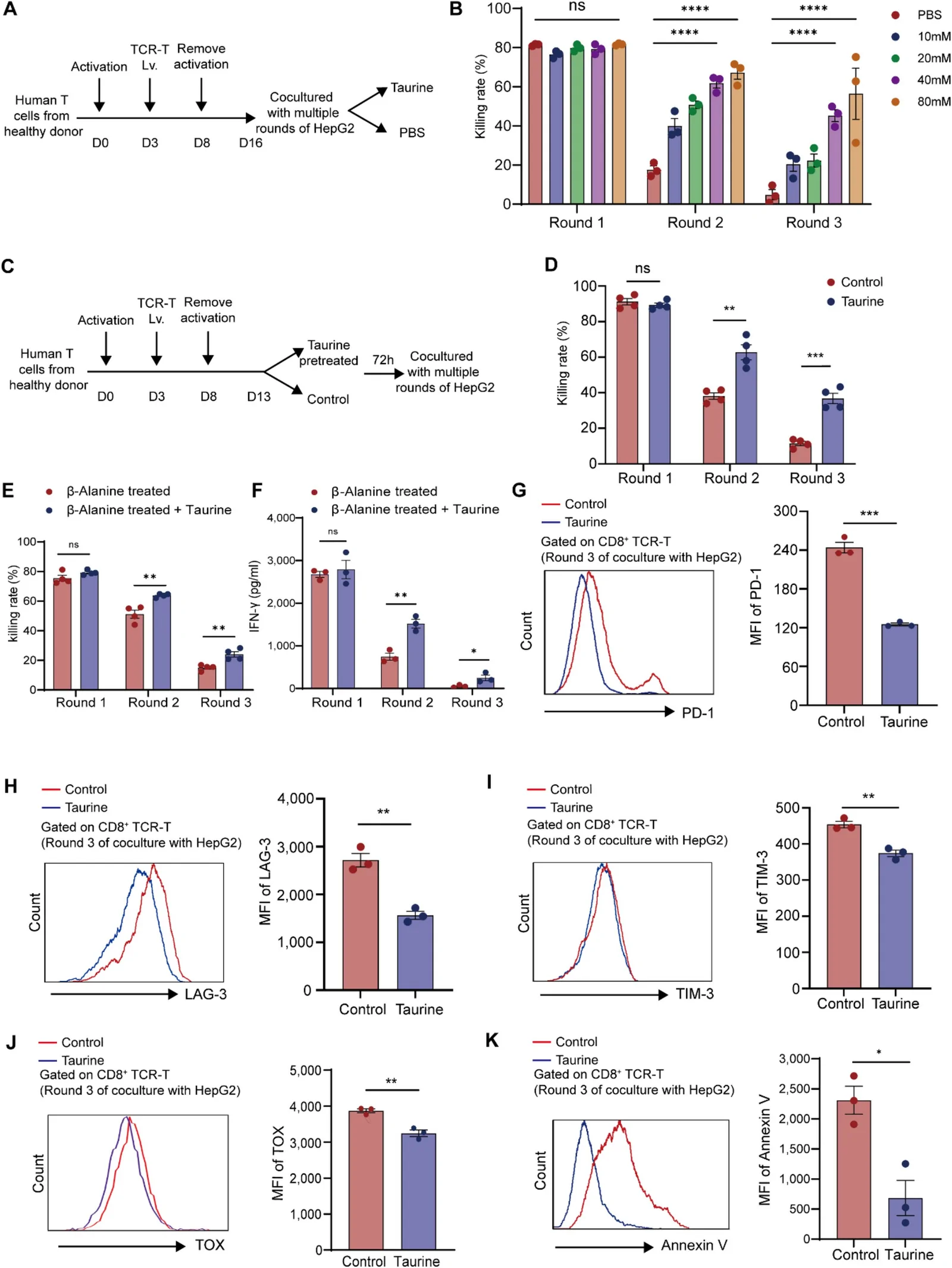
Taurine supplementation enhances TCR-T anti-HCC function in a concentration-dependent manner and reduces exhaustion and apoptosis. **A** The process illustrating the AFP-TCR-T generation, expansion, and coculture with HepG2. Briefly, T cells were isolated from healthy donors, transduced with AFP-TCR lentivirus and expanded ex vivo for 16 days. Then, the AFP-TCR-T were cocultured with multiple rounds of HepG2 in the presence or absence of varying concentration of taurine. **B** The bar graph showing cytotoxic activities of AFP-TCR-T supplemented with increasing concentrations of taurine during coculture with HepG2 (*n* = 3). PBS denotes the vehicle-control condition without taurine supplementation. **C** The schematic of taurine pretreatment for AFP-TCR-T in Fig. 2D-H. **D** The bar graph showing cytotoxic activities of AFP-TCR-T through challenge cycles with or without 40 mM taurine pretreatment (*n* = 4). **E** The bar graph showing cytotoxic activities of AFP-TCR-T pretreated with 100 mM β-alanine in the presence or absence of 40 mM taurine during sequential HepG2 tumor challenge cycles (*n* = 4). **F** The bar graph showing IFN-γ levels in the supernatants collected after coculture of AFP-TCR-T pretreated with 100 mM β-alanine in the presence or absence of 40 mM taurine with HepG2 cells during indicated challenge rounds (*n* = 4). **G**–**K** Representative histogram overlays and quantification of mean fluorescence intensity (MFI) of PD-1 (**G**), LAG-3 (**H**), TIM-3 (**I**), TOX (**J**), and Annexin V (**K**) in gated CD8^+^ AFP-TCR-T with or without 40 mM taurine pretreatment after three rounds of coculture with HepG2 cells (*n* = 3). Data were shown as mean ± SEM, *P < 0.05, **P < 0.01, ***P < 0.001, ****P < 0.0001, NS not significant

We next examined the effect of taurine on T-cell-mediated antitumor activity against HCC. Because taurine is highly accumulated intracellularly in leukocytes and has been reported to reach millimolar concentrations, taurine was tested at 10–80 mM as an exploratory in vitro conditioning range [34, 35 ]. Human AFP-TCR-T were generated and expanded ex vivo, and subjected to serial coculture with HepG2 cells in the presence of increasing concentrations of taurine (Fig. 3A). Taurine supplementation during coculture markedly enhanced the sustained cytotoxicity of AFP-TCR-T in a concentration-dependent manner, with the most prominent effects observed during the second and third rounds of coculture, whereas no significant difference was observed in the first round (Fig. 3B). In parallel, taurine promoted a stem-like phenotype in AFP-TCR-T, as indicated by an increased proportion of central memory T cells (T_CM_; CD62L^+^CD45RO^+^, Fig. S2A-B) and elevated TCF1 and LEF1 expression (Fig. S2C-D). Based on these results, 40 mM taurine was used for subsequent experiments, as cytotoxic activity plateaued at this level. To exclude potential direct effects of taurine on HepG2 cells, taurine supplementation was performed exclusively during the TCR-T expansion phase before coculture (Fig. 3C). Consistent with the initial findings, taurine-pretreated TCR-T exhibited enhanced antitumor activity compared with untreated controls, with greater tumor cell lysis during the second and third rounds of coculture (Fig. 3D).

To test whether taurine depletion–induced dysfunction was reversible, we treated β-alanine-impaired T cells with exogenous taurine. Taurine supplementation partially restored both cytotoxic activity and IFN-γ secretion in these compromised cells (Fig. 3E-F). Among gated CD8^+^ TCR-T cells after the third round of coculture, taurine supplementation reduced the expression of the exhaustion-associated markers PD-1, LAG-3, TIM-3, and TOX and decreased Annexin V staining, indicating reduced apoptotic susceptibility (Fig. 3G-K). These results demonstrate that taurine supplementation enhances the stem-like and sustained effector properties of AFP-TCR-T and can partially rescue taurine deficiency-associated functional impairment, exhaustion, and apoptosis.

### Taurine promotes in vivo antitumor function of AFP-TCR-T against HCC

**Fig. 4:**
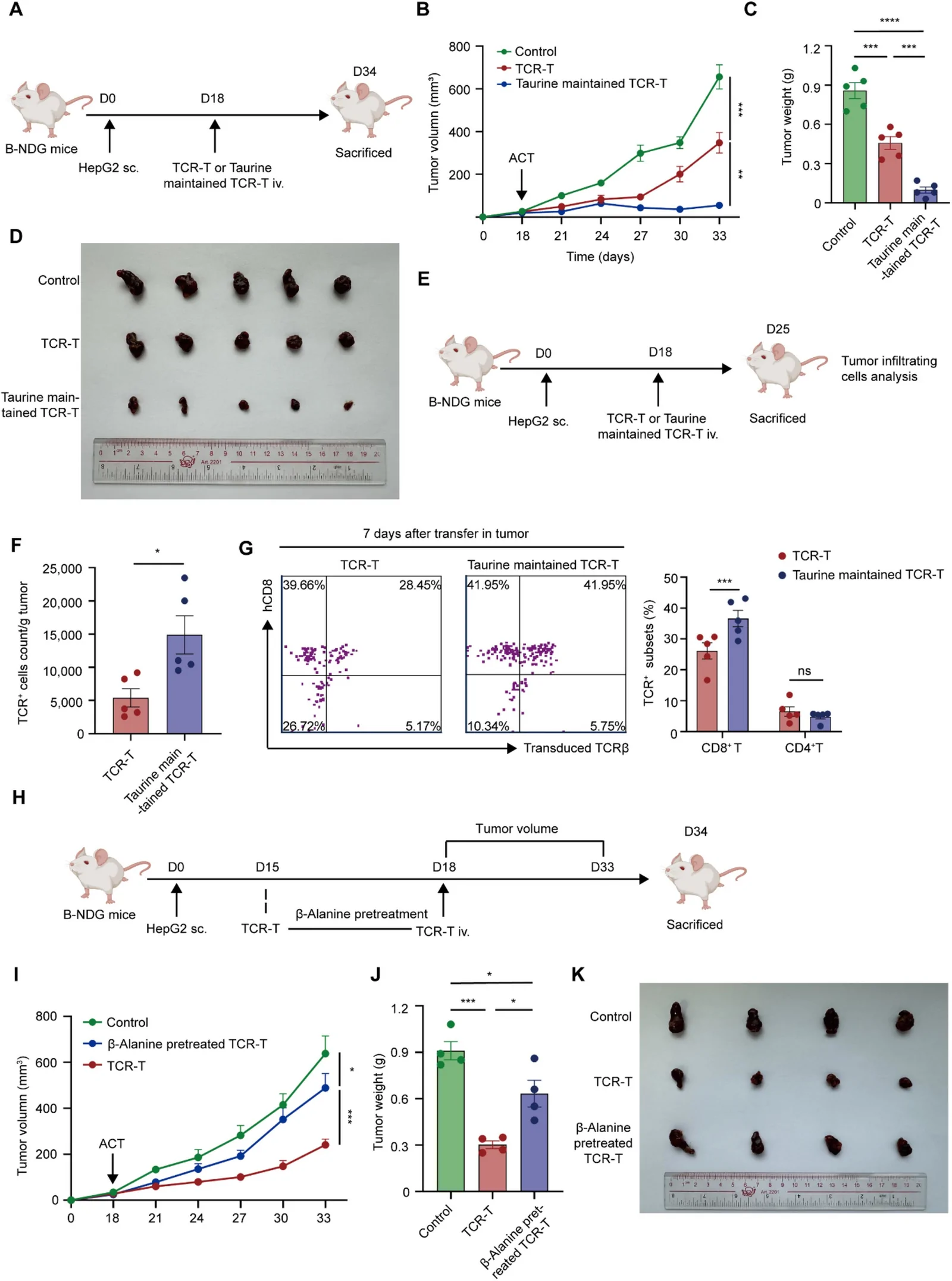
Taurine promotes the in vivo antitumor function of TCR-T against HCC. **A** The schematic of subcutaneous HepG2 model establishment and AFP-TCR-T or taurine-maintained AFP-TCR-T transfer process in B-NDG mice. **B** The line graphs depicting the tumor growth in each mouse of control, TCR-T, and taurine-maintained TCR-T groups after HepG2 implantation (*n* = 5 mice per group). Control indicates PBS-treated tumor-bearing mice. **C** The bar graph and **D** image showing the weight and volume of tumors isolated from mice in each group 33 days after HepG2 implant (*n* = 5 mice per group). **E** The schematic of subcutaneous HepG2 model establishment and AFP-TCR-T or taurine-maintained AFP-TCR-T transfer process in B-NDG mice in tumor-infiltrating TCR-T analysis experiment. **F** The bar graph showing tumor-infiltrating TCR^+^ cells counts in each gram tumor (*n* = 5 mice per group). **G** Scatter plots and the bar graph showing CD8^+^TCR^+^ and CD4^+^TCR^+^ subsets distribution in tumor after 7 days of AFP-TCR-T or taurine-maintained AFP-TCR-T transfer (*n* = 5 mice per group). **H** The schematic of subcutaneous HepG2 model establishment and AFP-TCR-T or β-alanine-pretreated AFP-TCR-T transfer process in B-NDG mice. **I** The line graph comparing the tumor growth of each mouse in untreated and β-alanine-pretreated AFP-TCR-T groups after HepG2 implantation (*n* = 4 mice per group). **J** The bar graph and **K** image showing the weight and volume of isolated tumors at the end of the experiment from tumor-bearing mice in each group (*n* = 4 mice per group). Data were shown as mean ± SEM, *P < 0.05, ***P < 0.001, ****P < 0.0001, NS not significant

To assess the in vivo relevance of these findings, we evaluated whether taurine pretreatment enhances AFP-TCR-T efficacy. In the first experiment, B-NDG mice bearing subcutaneous HepG2 tumors received taurine-pretreated or untreated AFP-TCR-T cells on day 18 after tumor implantation, whereas control mice received PBS vehicle (Fig. 4A). Tumor growth was monitored every 3 days, and taurine-pretreated AFP-TCR-T cells achieved markedly improved control of tumor progression (Fig. 4B). At day 34, tumors were harvested and weighed, with the taurine-pretreated AFP-TCR-T group showing the lowest tumor burden among all groups (Fig. 4C). Representative tumor images further supported these findings (Fig. 4D). In a separate in vivo experiment, we administered either taurine-pretreated or untreated AFP-TCR-T on day 18 after HepG2 cells implantation. Tumors were subsequently isolated on day 25 to assess tumor-infiltrating cells (Fig. 4E). Flow cytometry analysis revealed that the density of TCR^+^ cells per gram of tumor was elevated in the taurine pretreatment group compared to untreated TCR-T group (Fig. 4F). Meanwhile, the TCR^+^ subsets proportion within CD8^+^ T cells was significantly higher in the taurine-pretreated TCR-T group than the control TCR-T group (Fig. 4G), suggesting that taurine pretreatment enhanced the tumor infiltration of CD8^+^ AFP-TCR-T.

Next, we investigated the impact of taurine deficiency on T-cells in vivo antitumor function. B-NDG mice bearing subcutaneous HepG2 tumors were intravenously infused with untreated or β-alanine-pretreated AFP-TCR-T cells (Fig. 4H). Although both groups showed antitumor activity, tumor growth was more effectively controlled in mice receiving untreated AFP-TCR-T cells than in those receiving β-alanine-pretreated AFP-TCR-T cells (Fig. 4I). On Day 34 after HepG2 implantation, both tumor volume and weight were significantly lower in the mice receiving untreated AFP-TCR-T (Fig. 4J-K). These data collectively demonstrate that taurine pretreatment enhanced TCR-T infiltration within the tumor and effectively suppressed tumor growth, while taurine deficiency compromised TCR-T effector function against HCC in vivo.

### Taurine alters T-cell activation via regulating AKT phosphorylation

**Fig. 5:**
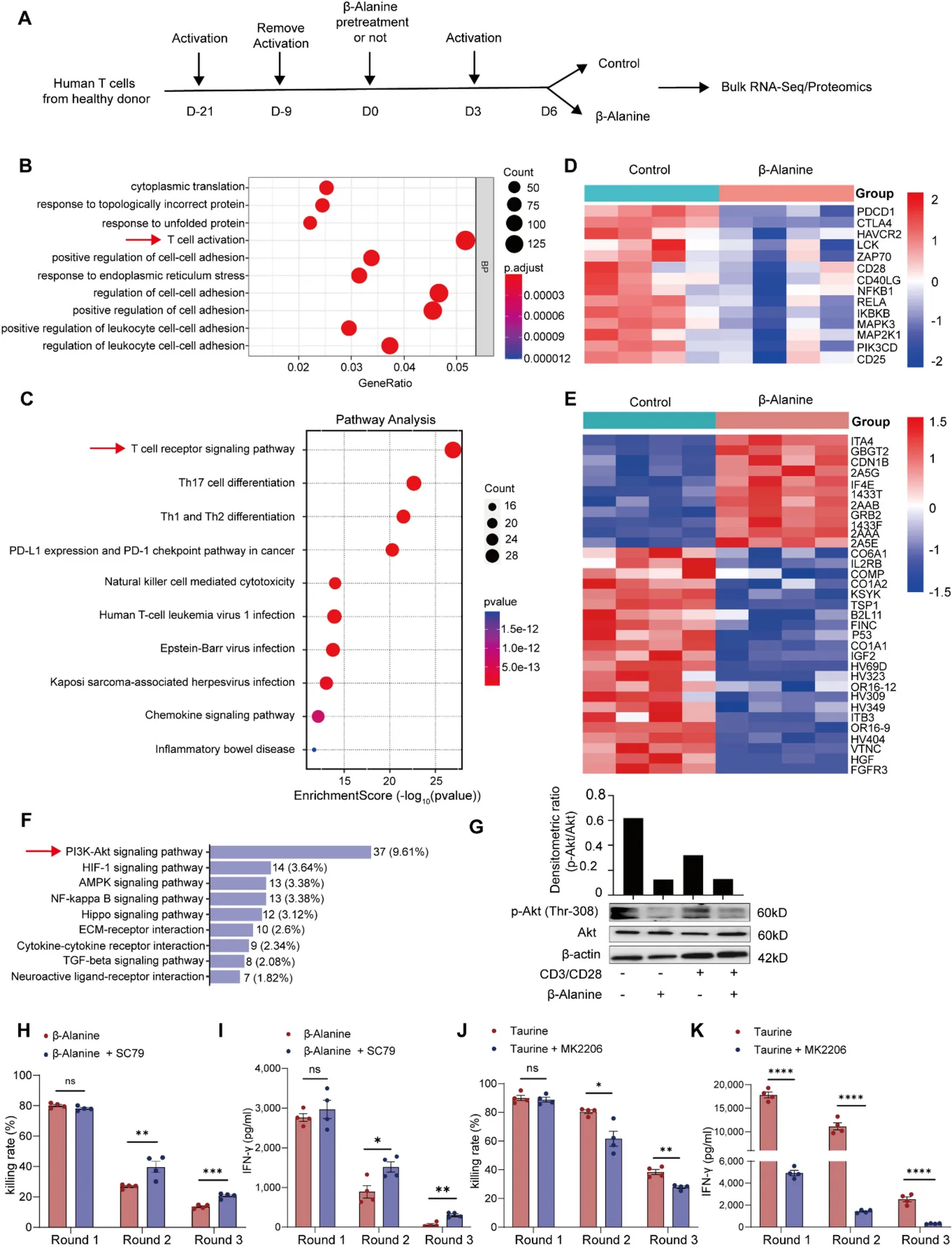
Taurine alters T-cell activation via regulating AKT phosphorylation. **A** The process of bulk RNA-Seq and proteomics analysis of activated T cells with or without β-alanine treatment. **B** The bubble plot displaying the KEGG enrichment pathway and **C** KOG enrichment pathway of differentially expressed genes between β-alanine-treated and untreated T cells. **D** The heatmap displaying differentially expressed genes associated with T-cell activation between β-alanine-treated and untreated T cells (*n* = 4). **E** The heatmap displaying differentially expressed proteins in the PI3K-AKT pathway between β-alanine-treated and untreated T cells (*n* = 4). **F** The bar graph showing the KEGG enrichment pathway of differentially expressed proteins between β-alanine-treated and control T cells. **G** Western blot analysis of phospho-AKT (Thr308), total AKT, and β-actin expression in T cells after CD3/CD28 activation with or without β-alanine treatment. Relative AKT phosphorylation was assessed by the phospho-AKT/total AKT grayscale intensity ratio. A representative blot from independent experiments with consistent results is shown. Grayscale intensity ratios were calculated from the displayed blot for descriptive purposes, and no statistical comparison was performed. **H** The bar graph showing cytotoxic activities of β-alanine-pretreated AFP-TCR-T during sequential HepG2 tumor challenge cycles in the presence or absence of the AKT activator SC79 (1 μM) (*n* = 4). **I** The bar graph showing IFN-γ levels in the supernatants collected after coculture of β-alanine-pretreated AFP-TCR-T with HepG2 cells during indicated challenge rounds in the presence or absence of SC79 (1 μM) (*n* = 4). **J** The bar graph showing cytotoxic activities of taurine-pretreated AFP-TCR-T during sequential HepG2 tumor challenge cycles in the presence or absence of the AKT inhibitor MK2206 (4 μM) (*n* = 4). **K** The bar graph showing IFN-γ levels in the supernatants collected after coculture of taurine-pretreated AFP-TCR-T with HepG2 cells during indicated challenge rounds in the presence or absence of MK2206 (4 μM) (*n* = 4). Data were shown as mean ± SEM, *P < 0.05, ***P < 0.001, ****P < 0.0001, NS not significant

To explore the mechanism underlying taurine-mediated regulation of T-cell function, we performed bulk RNA-Seq in taurine-depleted T cells (Fig. 5A). Genes downregulated by β-alanine were significantly enriched in T-cell activation and TCR signaling-related pathways by KOG and KEGG analyses (Fig. 5B-C), and key activation-associated transcripts were broadly suppressed (Fig. 5D). Consistently, proteomic analysis revealed significant enrichment of differentially expressed proteins in the PI3K-AKT signaling pathway (Fig. 5E-F). Western blot analysis suggested that β-alanine treatment reduced the phospho-AKT/total AKT ratio under both unstimulated and CD3/CD28-stimulated conditions, consistent with attenuated AKT phosphorylation (Fig. 5G). To determine whether taurine deficiency modulates T-cell function through the AKT signaling pathway, we employed the AKT activator SC79 and inhibitor MK2206. As expected, activation of AKT with SC79 partially rescued the impaired cytotoxic activity and IFN-γ secretion caused by taurine depletion (Fig. 5H-I), whereas inhibition of AKT with MK2206 attenuated the taurine-mediated enhancement of T-cell killing and IFN-γ secretion (Fig. 5J-K). Together, these findings support a model in which taurine promotes T-cell effector function through maintenance of AKT phosphorylation and PI3K-AKT signaling.

### Taurine deficiency induces hypermetabolic mitochondrial stress during T-cell activation

**Fig. 6:**
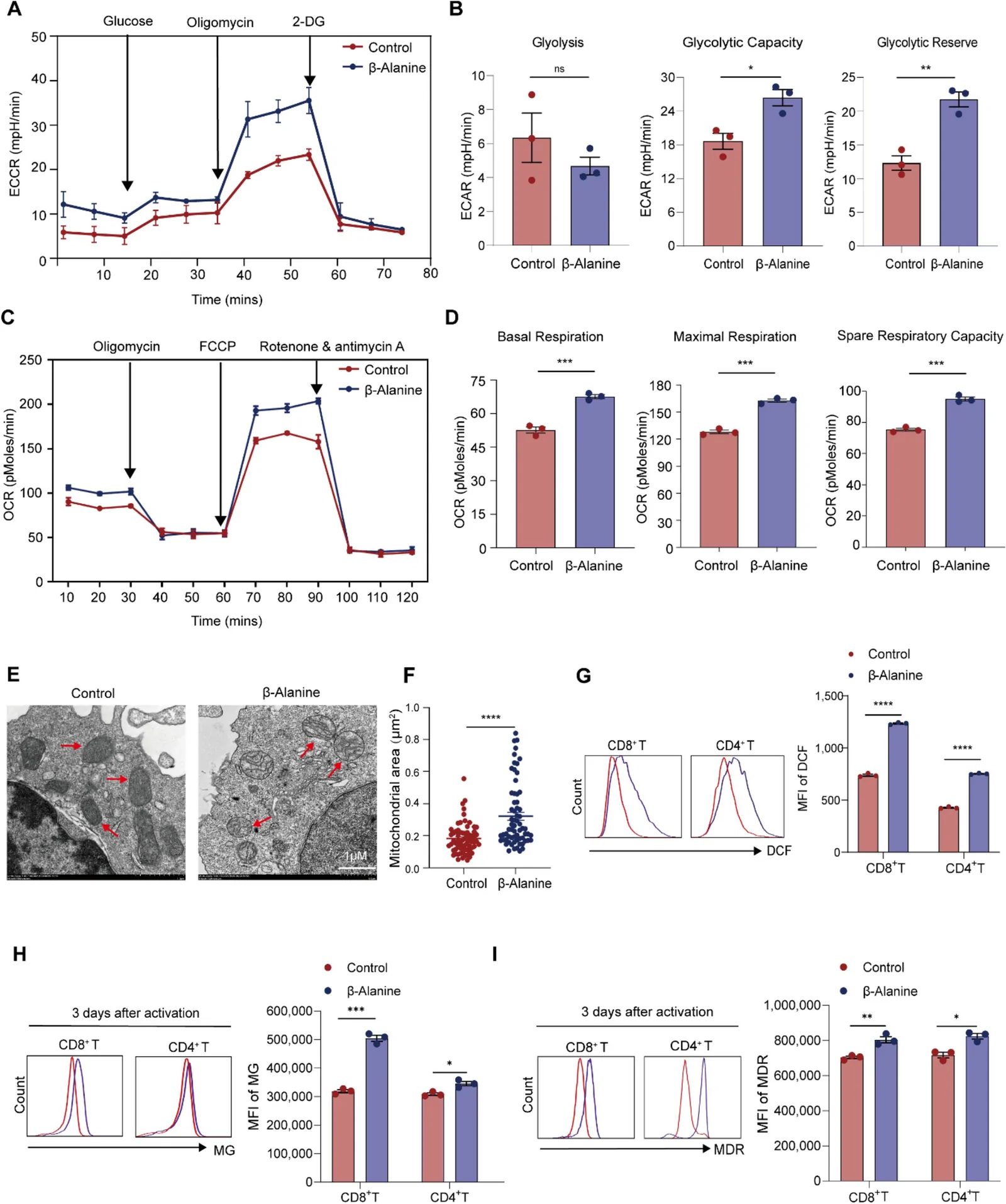
Taurine deficiency induces hypermetabolic mitochondrial stress during T-cell activation. **A** The line graph showing real-time extracellular acidification rate (ECAR) measurements of control and β-alanine-treated T cells after CD3/CD28 activation during glycolytic stress analysis with sequential injection of glucose, oligomycin, and 2-deoxy-D-glucose (2-DG) (*n* = 3). **B** Bar graphs quantifying glycolysis, glycolytic capacity, and glycolytic reserve derived from (**A**) (*n* = 3). **C** The line graph showing real-time OCR measurements of control and β-alanine-treated T cells after CD3/CD28 activation with metabolic inhibitors (FCCP, carbonyl cyanide 4-(trifluoromethoxy) phenylhydrazone; ROT/AA, rotenone and antimycin A) (*n* = 3). (D) Bar graphs quantifying basal OCR, maximal respiration, and spare respiratory capacity in (**C**) (*n* = 3). **E** Representative transmission electron microscopy (TEM) images showing mitochondrial morphology in control and β-alanine-treated T cells after activation. Red arrows indicate representative mitochondria (scale bar = 1 μm). **F** The scatter plot showing mitochondrial area distribution quantified from TEM images in control and β-alanine-treated T cells (*n* = 66 mitochondria). **G** Representative flow cytometry histogram overlays and quantification of DCFH-DA fluorescence, reflecting intracellular ROS levels, in CD8^+^ and CD4^+^ T cells with or without β-alanine treatment (*n* = 3). **H** Representative flow cytometry histogram overlays and quantification of MitoTracker Green (MG) MFI in CD8^+^ and CD4^+^ T cells 72 h after CD3/CD28 activation with or without β-alanine treatment (*n* = 3). **I** Representative flow cytometry histogram overlays and quantification of MitoTracker Deep Red (MDR) MFI in CD8^+^ and CD4^+^ T cells 72 h after CD3/CD28 activation with or without β-alanine treatment (n = 3). Data were shown as mean ± SEM, **P < 0.01, ***P < 0.001, ****P < 0.0001

Numerous studies have shown that AKT phosphorylation is correlated with cellular mitochondrial function and energy metabolism [36–38]. Given the reduction in AKT phosphorylation induced by β-alanine treatment, we therefore investigated whether taurine deficiency reshapes mitochondrial function and metabolic adaptation during T-cell activation. Seahorse analysis demonstrated that β-alanine-treated T cells exhibited significantly higher basal respiration, maximal respiration, and spare respiratory capacity than control cells (Fig. 6C-D). In addition, glycolytic capacity and glycolytic reserve were increased, although basal glycolysis was not significantly altered (Fig. 6A-B). Thus, taurine deficiency did not simply suppress cellular metabolism, but instead drove activated T cells into a hypermetabolic state.

Despite this apparent increase in bioenergetic activity, transmission electron microscopy revealed clear mitochondrial abnormalities in β-alanine-treated T cells, including swollen mitochondria with increased size, disrupted cristae architecture, thinning of the outer membrane, and signs of structural damage (Fig. 6E-F). In parallel, intracellular ROS levels were significantly elevated in both CD8^+^ and CD4^+^ T cells following β-alanine treatment (Fig. 6G), indicating enhanced oxidative stress after activation. MitoTracker Green (MG) and MitoTracker Deep Red (MDR) staining further showed increased mitochondrial content and enhanced membrane potential associated mitochondrial signal in β-alanine-treated cells (Fig. 6H-I). Taken together, these data indicate that taurine deficiency drives activated T cells into a maladaptive hypermetabolic state characterized by mitochondrial stress, oxidative damage, and structural abnormalities, which is associated with impaired effector function.

## Discussion

The efficacy of ACT against solid tumors, particularly HCC, depends not only on antigen specificity but also on the metabolic state and functional durability of transferred T cells. In this study, we identify taurine availability as an important regulator of T-cell activation and function, acting at least in part through the PI3K-AKT pathway. Taurine pretreatment enhanced the cytotoxic activity, intratumoral infiltration, and in vivo persistence of AFP-TCR-T, indicating that taurine may improve the therapeutic performance of ACT in HCC.

One notable observation was that intracellular taurine was dynamically regulated during T-cell activation and was also reduced in tumor tissues. Untargeted metabolomics and LC–MS validation showed that intracellular taurine declined during the early phase of CD3/CD28 stimulation, whereas SLC6A6 expression increased over time and reached its highest level at 48 h. In parallel, taurine abundance was lower in HCC tissues than in normal liver tissues in vivo. These findings suggest that taurine insufficiency may emerge under two biologically relevant conditions: during activation-associated metabolic remodeling of T cells and within the nutrient-constrained tumor microenvironment.

Taurine is widely recognized as an organic osmolyte and cytoprotective metabolite in mammalian cells [39]. During T-cell activation, rapid growth and metabolic reprogramming impose substantial demands on nutrient uptake, cell volume control, and biosynthetic balance [40]. Under these conditions, taurine may contribute to preservation of intracellular homeostasis. This interpretation is in line with earlier studies showing that *SLC6A6* expression protects effector T cells from activation-induced cell death and supports effective immune responses [23]. Viewed together with our data, these findings support a role for taurine in the metabolic adaptation of activated T cells rather than a merely passive intracellular presence.

Nutrient competition in the tumor microenvironment has become a recognized mechanism of immune suppression. Tumor-mediated methionine depletion, for example, including through *SLC43A2*, impairs histone methylation and suppresses IFN-γ production in T cells in the absence of exogenous supplementation [41]. Taurine availability may be shaped in a similar way. Previous studies have suggested that increased taurine uptake by tumor cells, associated with elevated *SLC6A6* expression, may reduce taurine availability and thereby impair T-cell function [24]. In the present study, β-alanine was used as a pharmacological tool to model taurine-limited conditions by competitively restricting taurine uptake [14]. Recent structural evidence has further clarified the molecular basis of human taurine transporter SLC6A6-mediated substrate uptake and inhibition, providing additional support for the use of β-alanine as a taurine-limiting tool [33]. Under this interpretation, the reciprocal effects of β-alanine and taurine supplementation consistently support a functional role for taurine availability in regulating T-cell state and antitumor activity. To minimize the possibility that taurine directly affected tumor cells, we adopted a T-cell preconditioning strategy in which taurine was administered before activation, without continuous exposure during coculture or adoptive transfer.

Our metabolic data further clarify the functional consequences of taurine limitation. Although β-alanine increased both OCR and ECAR during T-cell activation, these changes were not associated with improved T-cell fitness. Instead, the overall phenotype was more consistent with hypermetabolic stress, supported by increased glycolytic capacity and glycolytic reserve, elevated intracellular ROS, enlarged mitochondrial area, increased MG and MDR signals, and impaired long-term proliferative capacity. In this setting, increased OCR and ECAR are more plausibly interpreted as a maladaptive response than as evidence of beneficial metabolic optimization. By contrast, taurine appeared to support a more balanced metabolic state during activation, limiting oxidative stress while preserving functional fitness. Taurine is known to possess homeostatic and cytoprotective properties [14], which may help sustain effective activation while restraining exhaustion, apoptosis, and metabolic overload. In our system, taurine supplementation promoted a more stem-associated phenotype, enhanced effector cytokine production, and reduced apoptosis and exhaustion in TCR-T cells, with corresponding improvements in antitumor activity and intratumoral infiltration in HCC models. These findings are consistent with the emerging view that metabolic interventions can improve the efficacy of engineered T cell therapies [42, 43 ]. For example, CAR-T cells engineered to express arginine succinate synthase or ornithine transcarbamylase retain proliferative and cytotoxic capacity under arginine-restricted conditions [42]. Likewise, metabolic programs favoring fatty acid oxidation have been linked to enhanced T cell persistence, memory formation, and antitumor potential [43, 44 ]. Together, these studies support the idea that metabolic reprogramming is a key determinant of T-cell fate and a promising avenue for improving ACT durability in solid tumors [42, 43, 45 ].

Several limitations should be acknowledged. First, although β-alanine effectively models taurine-limited conditions, its effects may not be exclusively attributable to acute *SLC6A6* inhibition. Second, while our revised study now incorporates ECAR and ROS in addition to OCR, further multiparametric metabolic profiling will still be valuable to more fully define how taurine regulates T-cell metabolic plasticity under chronic stimulation. Third, although we demonstrated reduced taurine abundance in HCC tissues in vivo, genetic manipulation of *SLC6A6* in tumor cells was not performed in our model and will be important in future studies to directly test taurine competition within the tumor microenvironment. Fourth, all animal experiments in this study were performed exclusively with female mice. Given distinct physiological disparities between sexes, the conclusions drawn from the current work cannot be directly extended to male mice. Subsequent research utilizing male mice with optimized single-housing protocols is warranted to verify the universality of our results.

In summary, our study supports a model in which taurine availability links T-cell activation status, metabolic homeostasis, and antitumor durability. Taurine deficiency drives impaired proliferation, increased apoptosis, exhaustion, AKT suppression, and hypermetabolic mitochondrial stress, ultimately weakening the antitumor efficacy of AFP-TCR-T. By contrast, taurine supplementation preserves a more balanced, stem-associated, and functionally durable T-cell state and improves in vivo tumor control. These findings identify taurine availability as an important metabolic regulator of engineered T-cell efficacy and support metabolism oriented strategies for improving immunotherapy against solid tumors.

## Supplementary Information

Below is the link to the electronic supplementary material.

Supplementary file1 (DOCX 1116 KB)

## Data Availability

All experimental data generated or analyzed during this study are available from the corresponding authors upon reasonable request.
