# Supplementary material for: Taurine bolsters adoptive T cell therapy efficacy against hepatocellular carcinoma by reprogramming T cell function
Source: Cancer Immunol Immunother. 2026 Aug 6;75(11):268. doi: 10.1007/s00262-026-04503-y (PMC13731632; doi:10.1007/s00262-026-04503-y)
Supplement: Supplementary file 1 [file 262_2026_4503_MOESM1_ESM.docx]

**Supplementary material**

**Title:** Taurine bolsters adoptive T cell therapy efficacy against hepatocellular carcinoma by reprogramming T cell function

**Authors:** Zhiming Zhang^#，1,2^, Siwei Li^#，1,3^, Mengjie Li^#，1^, Li Gong^#，4^, Xinyu Lu^1^, Weikang Xu^5^, Anan Chen^1^, Yiyue Wang^1^, Changsheng He^4^, Guoheng Mo^1^, Yuting Xie^1^, Sian Yue^1^, Lei Huang^6^, Chou Yang^*,1^, Jinlin Hou^*,1^, Wei Zhu^*,1^

**1. Supplementary Methods**

**2. Figure S1~S2**

**Supplementary Methods**

**Flow-cytometry antibodies**

The antibodies used in this study include antibodies to human CD8 (Clone: SK1, BioLegend, San Diego, CA, USA), CD45 (Clone: 2D1, BioLegend), CD45RO (Clone: UCHL1, BioLegend), CD62L (Clone: DREG-56, BioLegend), PD-1 (Clone: EH12.2H7, BioLegend), TIM-3 (Clone: F38-2E2, BioLegend), LAG-3 (Clone: 7H2c65, BioLegend), TOX (Clone: W17072A, BioLegend), TCF1 (Clone:S33-966, BD Pharmingen^TM^), LEF1 (C12A5, Cell Signaling Technology), mouse TCRVβ8.3 (clone: H57-597, BioLegend), Annexin V (BioLegend) and mouse CD45.2 (Clone: 104, BioLegend).

**Untargeted metabolomics analysis**

The metabolomics of T cells was analyzed by Biotree Biomedical Technology Company (Shanghai, China). A total of 373 metabolites were identified by LC-MS and annotated. Briefly, the cell pellets (about 1×10^7^ cells) were taken, mixed with 1000 μL of extraction solution (MeOH : ACN : H2O, 2:2:1 (v/v)), the extraction solution contain deuterated internal standards, the mixed solution were vortexed for 30 s and incubated in liquid nitrogen for 1 min. The samples were then allowed to thaw at room temperature and vortexed for 30 s. This freeze–thaw cycle was repeated three times. Then the samples were sonicated for 10 min in 4 ℃ water bath, and incubated for 1 h at -40 ℃ to precipitate proteins. The samples were centrifuged at 12000 rpm (RCF=13800(×g), R= 8.6cm) for 15 min at 4 ℃. The supernatant was transferred to a fresh glass vial for analysis. LC-MS/MS analyses were performed using an UHPLC system (Vanquish, Thermo Fisher Scientific) with a Waters ACQUITY UPLC BEH Amide (2.1 mm × 50 mm, 1.7 μm) coupled to Orbitrap Exploris 120 mass spectrometer (Orbitrap MS, Thermo). The mobile phase consisted of 25 mmol/L ammonium acetate and 25 mmol/L ammonia hydroxide in water (pH = 9.75) (A) and acetonitrile (B). The auto-sampler temperature was 4 ℃, and the injection volume was 2 μL. The Orbitrap Exploris 120 mass spectrometer was used for its ability to acquire MS/MS spectra on information-dependent acquisition (IDA) mode in the control of the acquisition software (Xcalibur, Thermo). In this mode, the acquisition software continuously evaluates the full scan MS spectrum. The ESI source conditions were set as following: sheath gas flow rate as 50 Arb, Aux gas flow rate as 15 Arb, capillary temperature 320 ℃, full MS resolution as 60000, MS/MS resolution as 15000, collision energy: SNCE 20/30/40, spray voltage as 3.8 kV (positive) or -3.4 kV (negative), respectively.

The raw data were converted to the mzXML format using ProteoWizard and processed with an in-house program, which was developed using R and based on XCMS, for peak detection, extraction, alignment, and integration. Then an in-house MS2 database (BiotreeDB) was applied in metabolite annotation. The cutoff for annotation was set at 0.3.

**RNA-sequencing and analysis**

The RNA amount and purity of each sample was quantified using NanoDrop ND-1000 (NanoDrop, Wilmington, DE, USA). The RNA integrity was assessed by Bioanalyzer4200 (Agilent, CA, USA) with RIN number >6.0, Poly (A) RNA is purified from 0.1-4μg total RNA using VAHTS mRNA Capture Beads 2.0 N403-02 (Vazyme, China) using two rounds of purification. Then the poly(A) RNA was fragmented into small pieces using VAHTS Universal V8 RNA-seq Library Prep Kit for Illumina (Vazyme, China) under 94℃ 5min. Then the cleaved RNA fragments were reverse-transcribed to create the cDNA by VAHTS Universal V8 RNA-seq Library Prep Kit for Illumina (Vazyme, China), which were next used to synthesise U-labeled second-stranded DNAs with VAHTS Universal V8 RNA-seq Library Prep Kit for Illumina (Vazyme, China). An A-base is then added to the blunt ends of each strand, preparing them for ligation to the indexed adapters. Each adapter contains a T-base overhang for ligating the adapter to the A-tailed fragmented DNA. Single- or dual-index adapters are ligated to the fragments, and size selection was performed with VAHTS DNA Clean Beads. the ligated products are amplified with PCR by the following conditions: initial denaturation at 98℃ for 45 S; 14cycles of denaturation at 98℃ for 15 sec, annealing at 60℃ for 30 sec, and extension at 72℃ for 30 sec; and then final extension at 72℃ for 1 min. The average insert size for the final cDNA library was 300±50 bp. At last, we performed the 2×150bp paired-end sequencing (PE150) on an illumina Novaseq™ Xplus following the vendor's recommended protocol.

Fastp software (https://github.com/OpenGene/fastp) were used to remove the reads that contained adaptor contamination, low quality bases and undetermined bases with default parameter. Then sequence quality was also verified using fastp. We used HISAT2 (https://ccb.jhu.edu/software/hisat2) to map reads to the reference genome of Homo sapiens GRCh38. The mapped reads of each sample were assembled using StringTie (https://ccb.jhu.edu/software/stringtie) with default parameters. Then, all transcriptomes from all samples were merged to reconstruct a comprehensive transcriptome using gffcompare (https://github.com/gpertea/gffcompare/). After the final transcriptome was generated, StringTie and was used to estimate the expression levels of all transcripts. StringTie was used to perform expression level for mRNAs by calculating FPKM (FPKM = [total_exon_fragments / mapped_reads(millions) × exon_length(kB)]). The differentially expressed mRNAs were selected with fold change > 2 or fold change < 0.5 and with parametric F-test comparing nested linear models (p value < 0.05) by R package edgeR (<https://bioconductor.org/packages/release/bioc/html/edgeR.html>).

**Proteomic analysis**

Cells were lysed in lysis buffer (8 M urea, 1% SDS, 50 mM Tris-HCl, pH 8.0). Protein concentration was determined using a BCA assay (Fdbio science). For each sample, 100 µg of total protein was reduced with 5 mM DTT at 37 °C for 45 min and alkylated with 11 mM iodoacetamide (IAM) at room temperature for 15 min in the dark. The samples were then diluted to reduce urea concentration to <2 M, and digested overnight with sequencing-grade modified trypsin (Promega, V5280) at 37 °C. Adjust the pH of the digested peptides to 2-3 using 20%TFA, followed by desalting with C18 (Millipore, Billerica, MA) resin. Finally, determine the peptide concentration using a Pierce™ Quantitative Peptide Assay Kit with standards (Thermo Fisher).

About 200 ng peptides were separated on a reverse-phase C18 analytical column (75 μm × 15 cm, 1.6 μm particle size) using a linear gradient of 5–25% acetonitrile with 0.1% formic acid within 20 minutes at a flow rate of 0.5μL/min. MS raw data were analyzed using DIA-NN (v1.8.1) with library-free method. the uniprotkb_proteome_UP000005640_human_82493_20240528.fasta database (A total of 82493 sequences) was uesed to creat a spectra library with deep learning algrithms of neural networks. the option of MBR (Match Between Runs) was employed to create a spectral library from DIA data and then reanlyse using this library. FDR (false discovery rate) of search results was adjusted to < 1% at both protein and precursor ion levels, the remaining identifications were used for further quantification analysis.

**Figure S1**

**Figure S1 The metabolomics profile of T cells activation.** (A) The PCA analysis of metabolomics activated and non-activated T cells. (B) The volcano plot of differential metabolites between activated and non-activated T cells. (C-D) The differential metabolite pathway detected in negative ion mode. (E-F) The differential metabolite pathway detected in positive ion mode.

**Figure S2**

**Figure S2 Taurine supplementation promotes AFP-TCR-T stemness.** (A) The gating strategy of CD8^+^ TCR-T cells after coculture with HepG2 detected by flow-cytometry. (B) The CD45RO and CD62L expression of CD8^+^ TCR-T after 3-round coculture with HepG2 were measured by flow cytometry. The representative plots and percentage of central-memory T cell subsets (T_cm_) were shown. (n = 3) (C–D) Representative histogram overlays and quantification of TCF1 (C) and LEF1 (D) MFI in CD8⁺ AFP-TCR-T cells after multiple rounds of coculture with HepG2 cells, with or without taurine pretreatment (n = 3). Data were shown as mean ± SEM, ***P* < 0.01, ****P* < 0.001.
